# A Prospective Cohort Study on the Prevalent and Recurrent Tuberculosis Isolates Using the MIRU-VNTR Typing

**DOI:** 10.3389/fmed.2021.685368

**Published:** 2021-09-14

**Authors:** Beibei Qiu, Bilin Tao, Qiao Liu, Zhongqi Li, Huan Song, Dan Tian, Jizhou Wu, Zhuchao Wu, Mengyao Zhan, Wei Lu, Jianming Wang

**Affiliations:** ^1^Department of Epidemiology, Center for Global Health, School of Public Health, Nanjing Medical University, Nanjing, China; ^2^Department of Chronic Communicable Disease, Center for Disease Control and Prevention of Jiangsu Province, Nanjing, China

**Keywords:** tuberculosis, molecular epidemiology, genotyping, recurrence, reactivation, reinfection, Beijing family

## Abstract

The study aims to describe the clustering characteristics of *Mycobacterium tuberculosis* (*M.tb*) strains circulating in eastern China and determine the ratio of relapse and reinfection in recurrent patients. We recruited sputum smear-positive pulmonary tuberculosis cases from five cities of Jiangsu Province, China, during August 2013 and December 2015. Patients were followed for the treatment outcomes and recurrence based on a cohort design. *M.tb* strains were isolated and genotyped using the 12-locus MIRU-VNTR. The Beijing family was identified by the extended Region of Difference (RD) analysis. The Hunter-Gaston Discriminatory Index (HGDI) was used to judge the resolution ability of MIRU-VNTR. The odds ratio (OR) together with 95% confidence interval (CI) were used to estimate the strength of association. We performed a cluster analysis on 2098 *M.tb* isolates and classified them into 545 genotypes and five categories (I, 0.19%; II, 0.43%; III, 3.34%; IV, 77.46%; V, 18.59%). After adjusting for potential confounders, the Beijing family genotype (OR = 118.63, 95% CI: 79.61–176.79, *P* = 0.001) was significantly related to the dominant strain infections. Patients infected with non-dominant strains had a higher risk of the pulmonary cavity (OR = 1.39, 95% CI: 1.01–1.91, *P* = 0.046). Among 37 paired recurrent cases, 22 (59.46%) were determined as endogenous reactivation, and 15 (40.54%) were exogenous reinfection. The type of *M.tb* strains prevalent in Jiangsu Province is relatively single. Beijing family strains infection is dominant in local tuberculosis cases. Endogenous reactivation appears to be a major cause of recurrent tuberculosis in Eastern China. This finding emphasizes the importance of case follow-up and monitoring after the completion of antituberculosis treatment.

## Introduction

Tuberculosis (TB) is a common chronic infectious and consumptive disease that damages the organs of the whole body. It has been ranked as one of the top 10 causes of death and the greatest killer of a single infectious disease in the world (1). According to the 2020 Global Tuberculosis Report (https://www.who.int/), an estimated 10.0 million (range 9.0–11.1 million) people fell ill with TB in 2019, a number that has been relatively stable in recent years. Thirty high TB burden countries, including India, China, Indonesia, Philippines, and other countries, account for 87% of the global cases.

Patients with recurrent TB are less likely to complete treatment and suffer higher fatality than those with the first episode of TB (2). Recurrence of TB can be due to either relapse of an initial infection or exogenous reinfection with new *Mycobacterium tuberculosis (M.tb)* strains (3). The causes of such two types of recurrence are different (4). Poor treatment adherence (5), positive sputum smear, pulmonary cavity (6), drug resistance (7), old age (8), or Beijing family *M.tb* strains (9) are the risk factors of endogenous reactivation, while human immunodeficiency virus (HIV) infection (10) and diabetes (11) can increase the risk of exogenous reinfection. Because TB recurrence is usually associated with drug resistance, it is reasonable to distinguish the types of TB recurrence and to understand the mechanism of endogenous reactivation and exogenous reinfection.

With the development of molecular biological techniques, we can genotype the strains of *M.tb* that broke out circulating in a particular area to analyze the epidemiological characteristics of the pathogen and clarify factors related to the transmission. Molecular genotyping methods, such as IS6110 restriction fragment length polymorphism (IS6110-RFLP), spacer oligonucleotide typing (Spoligo-typing), mycobacterial interspersed repetitive units (MIRU), and the variable number of tandem repeats (VNTR), have been applied to genotype TB isolates (12). TB genotyping helps to identify the outbreak and epidemic process, trace the source of infection, establish a phylogenetic relationship and identify patients whose disease is the result of reactivation of an infection that was acquired in the past or involved in the same chain of recent transmission (13). MIRU-VNTR is a PCR-based typing method that calculates the size and repeated number of units in each locus by amplifying mycobacterial interspersed repetitive units (12). Various combinations of MIRU-VNTR loci have been used to give better differentiation of *M.tb* strains. The typical combinations include 12, 15, and 24-loci. The 15 and 24-loci of MIRU-VNTR include the previous 12 loci, together with several additional high-resolution loci (14). However, the allelic diversity of loci can vary between areas or between *M.tb* complex species (15).

Jiangsu is an area with a higher population density and a province with higher TB mobility in Eastern China. To describe the molecular typing characteristics and epidemic trends of *M.tb* is of great significance for the targeted disease prevention and control strategies. In the current study, we selected five cities in Jiangsu Province as the study sites, continuously recruited TB cases as study subjects, and genotyped clinical isolates of *M.tb* using the MIRU-VNTR. TB patients were followed to record the treatment outcomes and monitor the recurrence. This study had two purposes. Firstly, we attempted to analyze the clustering characteristics of TB isolates and related factors. Secondly, we aimed to determine the ratio of relapse and reinfection in recurrent patients and explore the molecular types of recurrent infections.

## Materials and Methods

### Patients

We selected Lianyungang, Xuzhou, Nantong, Changzhou, and Taizhou in Jiangsu Province, China, as the study sites. Patients diagnosed during August 2013 and December 2015 were recruited as study subjects and followed to record the disease's recurrence. We defined the recurrence as a TB episode occurring within the study period after treatment completion or cure of a previous episode. The interval of two disease episodes should be longer than 1 year. Newly diagnosed sputum smear-positive TB cases were included. Extrapulmonary TB or HIV-positive cases were excluded.

### Samples Collection

Sputum smear microscopy test, culture, and strain identification were performed following the guideline of the Tuberculosis Diagnosis Laboratory Test Regulations of China Tuberculosis Association. *M.tb* strains were harvested from the Lowenstein-Jensen medium and preserved in the freezer at −80°C. The DNA of *M.tb* strains was extracted by a modified CTAB (hexadecyltrimethylammonium bromide) method. In brief, the colony of the 10 ul quantitative inoculation ring was scraped in the biosafety cabinet, suspended in the EP tube equipped with 200 ul sterilized TE, and inactivated in a water bath at 80°C for 30 min. The inactivated bacteria were boiled at 100°C for 10 min and centrifuged with 12,000 r/min for 15 min. The DNA template was obtained by absorbing the supernatant and stored at −20°C.

### Drug Sensitivity Test

A drug sensitivity test was carried out by using the proportional method (16, 17). Samples to be tested were diluted to 10^−2^ and 10^−4^ g/L bacterial suspensions. We dipped 1 ring (0.01 ml) of the bacterial suspensions with a 22 standard wire gauge (SWG) quantitative inoculation ring and inoculated them on the surface of the control and drug-containing medium. The bacterial liquid was dispersed on the slope of the culture medium as evenly as possible, and the final inoculation amount was 10^−4^ mg and 10^−6^ mg, respectively. After inoculation, the culture medium was placed at 37°C, and the number of colonies was read after 4 weeks to calculate the resistance percentage, where ≤1% was judged as sensitive, and >1% was considered resistant.

### Beijing Family Identification

The Beijing family isolates were identified by extended genotyping Region of Difference (RD) analysis. The detection of the genomic deletions RD105 was done by PCR using primers previously described (18). If the strain had the RD105 genomic deletion, it was defined as the Beijing family strain.

### MIRU-VNTR Genotyping

Although there are various combinations of MIRU-VNTR loci, no specific set of loci have been agreed upon as a standard. Considering the time-consuming and economic cost, some researchers recommended using 12-loci MIRU-VNTR. Besides, the resolution of 12-loci MIRU-VNTR is relatively high, and there may be over-clustering by 15- or 24-loci MIRU-VNTR for Beijing strains (19). Thus, in this study, we applied the 12-loci MIRU-VNTR to genotype the strains isolated from recurrent patients. Each MIRU locus was amplified individually with primers specific for sequences flanking the MIRU units (Supplementary Table S1). In the current study, we used a set of 12 VNTR loci (20, 21). The PCR reaction mixture for all loci was listed in Supplementary Table S2. The amplification profile consisted of pre-denaturation at 94°C for 5 min, followed by 25 cycles of 94°C for 45 s, 60°C for 50 s, and 72°C for 60 s, and 72°C extensions for 10 min. We used the H37Rv strain as a standard control to determine the size of the amplified fragment.

### Statistical Analysis

Cluster analyses were performed using R software for Windows version 3.5.3 (https://www.r-project.org/) with “hclust,” “phangorn,” “ape,” and “tidyverse” package. We used the MIRU-VNTRplus web application (https://www.miru-vntrplus.org) to compare the strains with the reference strains. We used iTOLs (https://itol.embl.de/) to modify and style the phylogenetic tree. The Hunter-Gaston Discriminatory Index (HGDI) (22) was used to judge the resolution ability of MIRU-VNTR. $HGDI=1-\frac{1}{N\left(N-1\right)}\overset{s}{\underset{j=1}{\sum }}nj\left(nj-1\right)$. *N* stands for the total number of strains, *nj* is the number of strains with the *j*th genotype, and *s* is the number of different genotypes at the MIRU-VNTR loci. HGDI ≥0.60 was judged as a high polymorphism, 0.30 ≤ HGDI < 0.60 showed a medium polymorphism, and HGDI <0.30 indicated a low polymorphism. We applied a logistic regression model to explore factors related to dominant and non-dominant isolates and used the Fisher exact test to compare the characteristics of recurrent types using SPSS software (version 25.0, IBM Corporation, Armonk, NY). A two-tailed *P*-value of <0.05 was regarded as statistically significant.

## Results

### Data Collection

**Figure 1:**
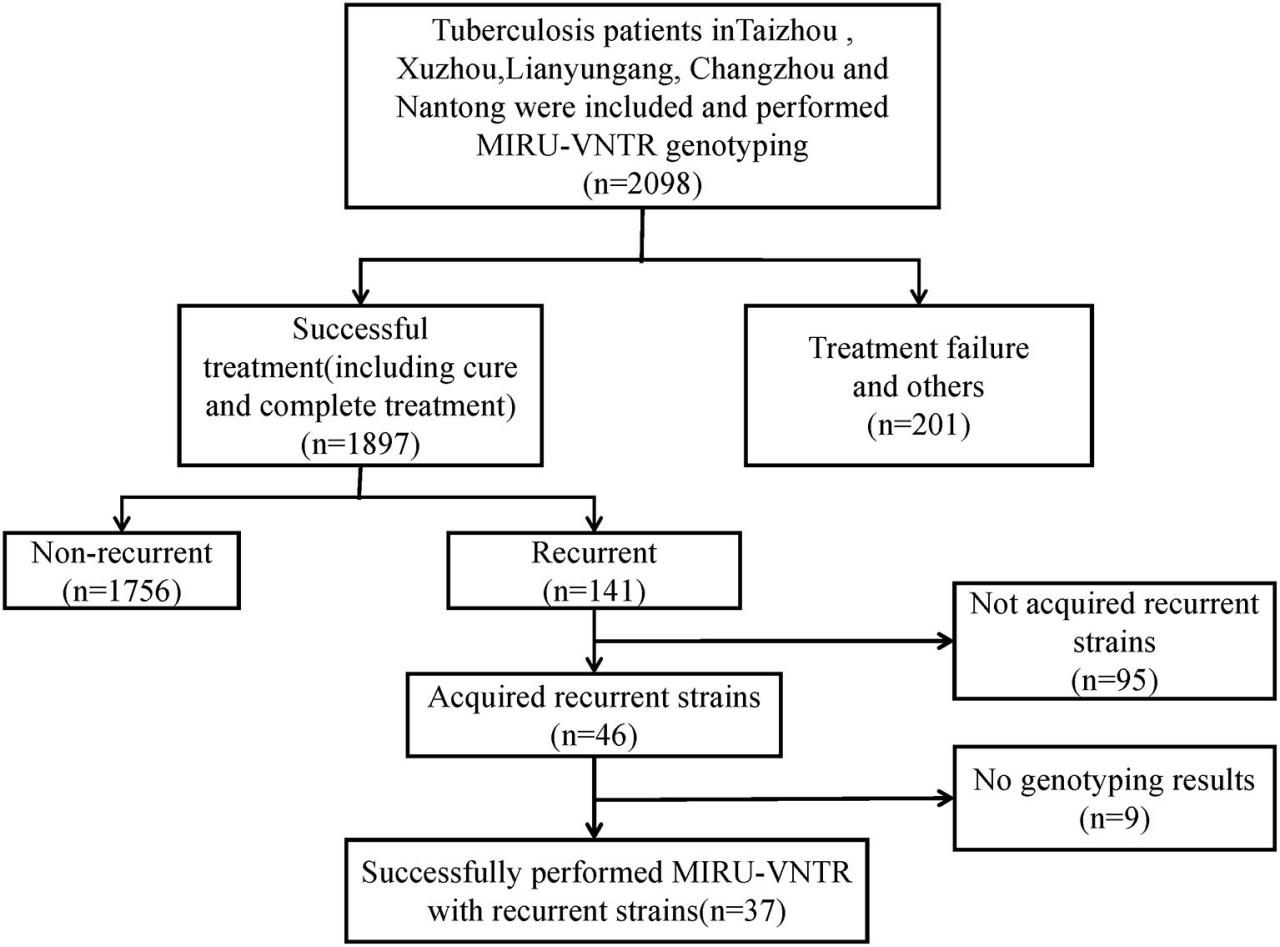
Eligibility and enrollment of included participants. MIRU-VNTR, Mycobacterial Interspersed Repetitive Units-Variable Number of Tandem Repeats; MDR-TB, Multi-drug resistant tuberculosis.

We recruited 2,098 TB cases from August 2013 to December 2015. After the standard antituberculosis therapy, 1,897 patients were treated successfully (including cured and completed treatment), the other 201 patients were including death, treatment failure, adverse reactions, refuse treatment, change treatment regime, MDR-TB treatment, and others. After follow-up on cases that were cured and completed treatment, we observed 141 recurrent cases. After excluding contaminated isolates, incomplete retention of primary and recurrent isolates, and samples with failed DNA extraction, 37 pairs of recurrent cases were used for MIRU-VNTR genotyping comparison (Figure 1).

### Cluster Analysis of All Isolates

**Figure 2:**
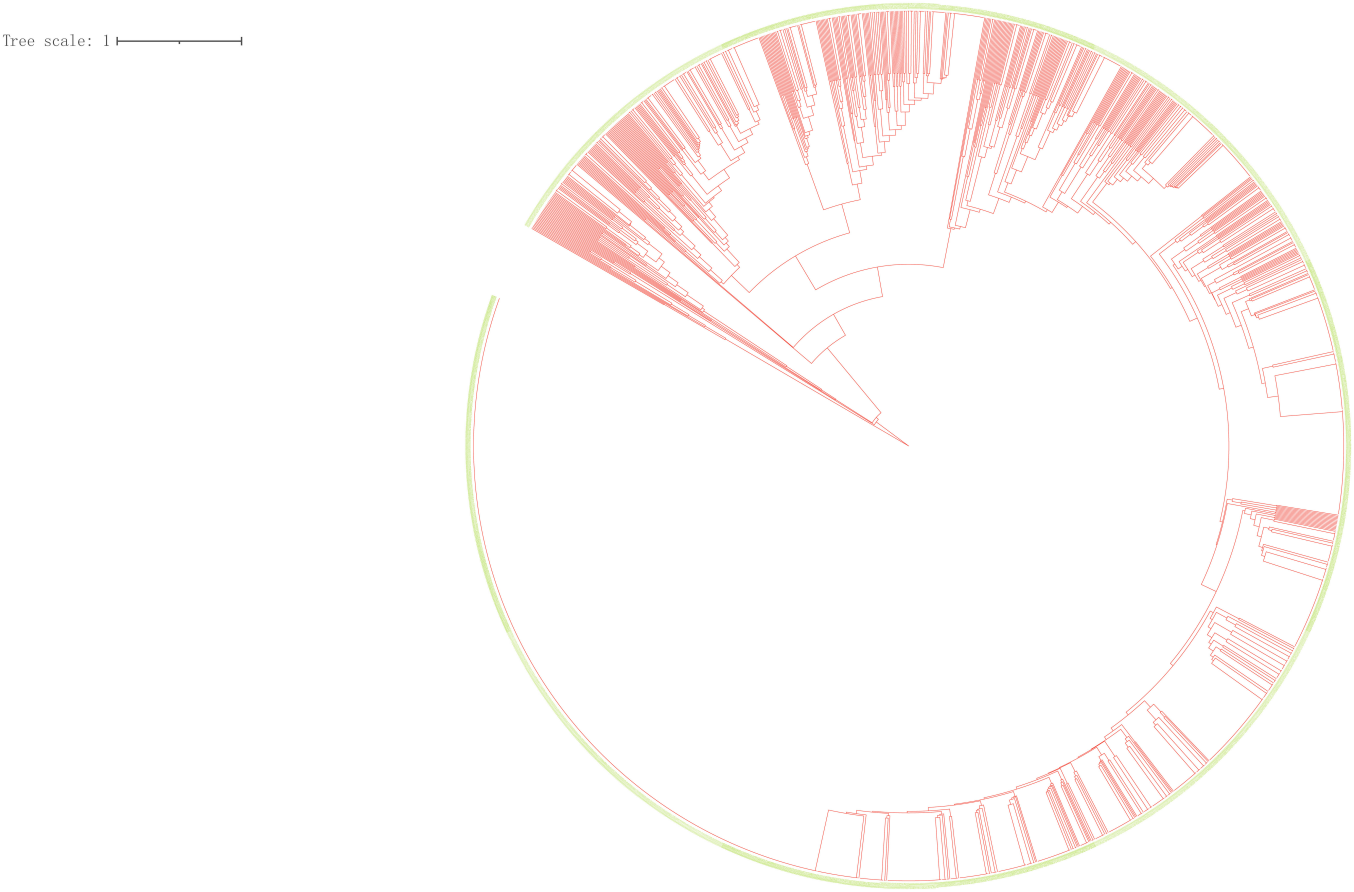
Clustering tree of 2,098 tuberculosis isolates. Red represents the branches of the phylogenetic tree, and green means the ID of the strains.

We performed a cluster analysis on 2,098 isolates to observe the classification tree and clustering (Figure 2). Red represents the branches of the phylogenetic tree, and green means the ID of the strains. All isolates could be divided into 545 genotypes, including 156 clustered genotypes. Three hundred eighty-nine isolates had independent genotypes. Among 1,709 clustered isolates, the largest cluster had 586 isolates (27.93%). We further divided 2,098 isolates into five categories (I, 4, 0.19%; II, 9, 0.43%; III, 70, 3.34%; IV, 1,625, 77.46%; V, 390, 18.59%). We defined the strains which belonged to a category that exceeded 70% of the total number of strains as the dominant strain. Infection with dominant strains was related to Beijing family genotype (OR = 115.95, 95% CI: 78.21–171.91, *P* = 0.001) and drinking (OR = 0.67, 95% CI: 0.53–0.86, *P* = 0.002). After adjusting for potential confounders, such as sex, age, smoking, drinking, Beijing family genotype, contact history, MDR-TB, treatment outcomes, and pulmonary cavity, Beijing family genotype (OR = 118.63, 95% CI: 79.61–176.79, *P* = 0.001) remained significant. Patients with the pulmonary cavity (OR = 1.39, 95% CI: 1.01–1.91, *P* = 0.046) had a higher risk of infection with non-dominant strains (Table 1).

**Table 1:** Factors associated with dominant and non-dominant strains distinguished by MIRU-VNTR Genotypes.

**Risk factors**		***N***	**Dominant strains**		**Non-dominant strains**		**Univariate analysis**		**Multivariate analysis**	
			***n***	**%**	***n***	**%**	**cOR (95% CI)**	***P*** **-value**	**aOR (95% CI)**	***P*** **-value**
Sex	Male	1,631	1,252	77.05	379	80.13	0.83 (0.65–1.07)	0.157	1.00 (0.66–1.53)	0.996
	Female	467	373	22.95	94	19.87	Reference		Reference	
Age (years)	<=25	316	252	15.51	64	13.53	Reference		Reference	
	26–54	685	534	32.86	151	31.92	0.90 (0.65–1.25)	0.522	1.10 (0.67–1.80)	0.722
	≥55	1,097	839	51.63	258	54.55	0.83 (0.61–1.12)	0.223	0.98 (0.61–1.57)	0.931
Smoking	Yes	831	634	39.02	197	41.65	0.90 (0.73–1.10)	0.303	1.23 (0.85–1.77)	0.280
	No	1,267	991	60.98	276	58.35	Reference		Reference	
Drinking	Yes	389	278	17.11	111	23.47	0.67 (0.53–0.86)	0.002	0.72 (0.47–1.10)	0.126
	No	1,709	1,347	82.89	362	76.53	Reference		Reference	
Beijing family	Yes	1,727	1,591	97.91	136	28.75	115.95 (78.21–171.91)	0.001	118.63 (79.61–176.79)	0.001
	No	371	34	2.09	337	71.25	Reference		Reference	
Contact history	Yes	159	118	7.26	41	8.67	0.83 (0.57–1.20)	0.310	0.93 (0.52–1.66)	0.801
	No	1,939	1,507	92.74	432	91.33	Reference		Reference	
MDR-TB	Yes	87	73	4.49	14	2.96	1.54 (0.86–2.76)	0.144	1.16 (0.48–2.79)	0.742
	No	2,011	1,552	95.51	459	97.04	Reference		Reference	
Recurrence	No	1,756	1,358	83.57	398	84.14	Reference		Reference	
	Yes	141	114	7.02	27	5.71	1.24 (0.80–1.91)	0.336	0.97 (0.51–1.82)	0.920
	Others	201	153	9.41	48	10.15	0.93 (0.66–1.32)	0.697	0.82 (0.47–1.45)	0.499
Pulmonary cavity	Yes	987	751	46.22	236	49.89	Reference		Reference	
	No	1,111	874	53.78	237	50.11	1.16 (0.94–1.42)	0.158	1.39 (1.01–1.91)	0.046

*Dominant strains are defined as a category that exceeds 70% of the total number of strains. MIRU-VNTR, Mycobacterial Interspersed Repetitive Units—Variable Number of Tandem Repeats; MDR-TB, Multi-drug resistant tuberculosis; cOR, Crude odds ratio; aOR, Adjusted odds ratio; CI, Confidence interval*.

For 12 sites used for genotyping, MIRU 26 had a high polymorphism (HGDI = 0.6262), MIRU 10, 31, 39, and 40 had a moderate polymorphism (0.30 ≤ HGDI < 0.60), while MIRU 2, 4, 16, 20, 23, 24, and 27 had a low polymorphism (HGDI < 0.30) (Table 2).

**Table 2:** Resolution of each polymorphic site for 2,098 isolates.

**Index**	**MIRU-VNTR locus**											
	**2**	**4**	**10**	**16**	**20**	**23**	**24**	**26**	**27**	**31**	**39**	**40**
Copy number												
0												
1	9	59	52	15	37	3	2,092	48	7	3	15	89
2	2,073	1,836	497	151	2,054	6	6	10	227	32	414	264
3	16	27	1,466	1,890	7	5		62	1,836	328	1,617	1,619
4		122	62	42		18		86	27	192	50	114
5		40	16			2,035		305	1	1,460	2	12
6		4	1			25		230		74		
7		3				5		1,215		7		
8		3	1			1		96		2		
9		3	3					39				
10		1						7				
HGDI	0.0236	0.2296	0.4543	0.1829	0.0412	0.0589	0.0057	0.6262	0.2224	0.4816	0.3666	0.3841

*MIRU-VNTR, Mycobacterial Interspersed Repetitive Units—Variable Number of Tandem Repeats; HGDI, Hunter-Gaston, Discriminatory Index*.

### Cluster Analysis of Recurrent Cases

**Figure 3:**
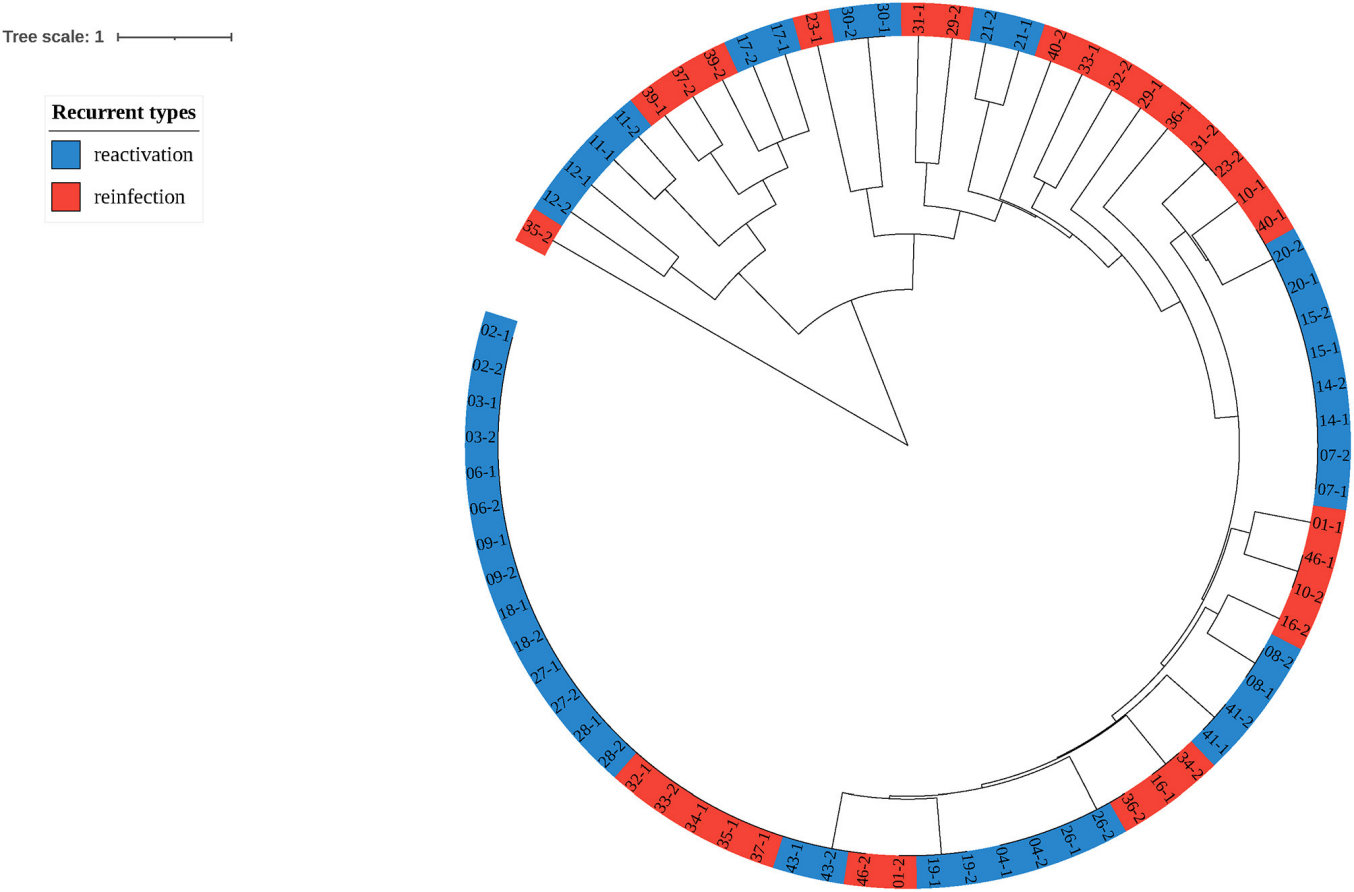
Clustering tree of 37 pairs of recurrent tuberculosis isolates. The red label represents reinfection of tuberculosis, while the blue represents reactivation.

To explore the proportion of relapse and reinfection among recurrent cases, we compared the MIRU-VNTR genotypes of 37 paired isolates. The copy number and HGDI index were shown in Table 3. We have also listed the total copy numbers of 37 paired recurrent strains and Beijing family genotypes obtained by the RD105 test in Supplementary Table S3. Endogenous or reactivation paired cases have the same lineages. The polymorphism of MIRU 10, 26, 31, and 39 kept at a moderate level (0.30 ≤ HGDI < 0.60), while MIRU 2, 4, 16, 20, 23, 24, 27 and 40 had low polymorphisms (HGDI < 0.30). The isolates with similar characteristics were grouped into one genotype by cluster analysis (Figure 3). Red and blue labels represent reinfection and reactivation, respectively.

**Table 3:** Resolution of each polymorphic site for 37 paired cases.

**Index**	**MIRU-VNTR locus**											
	**2**	**4**	**10**	**16**	**20**	**23**	**24**	**26**	**27**	**31**	**39**	**40**
Copy number												
0					1			1	1			
1		1	2	2			74			2		
2	74	66	15	4	73				6	1	14	4
3		1	56	68		1		3	66	8	59	65
4		4	1					3	1	3	1	5
5		2				71		9		55		
6								11		5		
7						2		46				
8								1				
HGDI	0	0.2033	0.3906	0.1540	0.0270	0.0796	0	0.5809	0.2002	0.4317	0.3328	0.2240

*MIRU-VNTR, Mycobacterial Interspersed Repetitive Units—Variable Number of Tandem Repeats; HGDI, Hunter-Gaston, Discriminatory Index*.

Among 37 paired recurrent cases, 22 (59.46%) were regarded as endogenous reactivation, and 15 (40.54%) were exogenous reinfection. In the endogenous relapse, males (86.36%) and the elderly (68.18%) accounted for a higher proportion than exogenous reinfection. The proportion of tobacco smoking history was also higher in patients with endogenous relapse (50.00%) than in patients with exogenous reinfection (26.67%). Patients with pulmonary cavities were more likely to suffer from exogenous reinfection (66.67%) than endogenous relapse (45.45%). Recurrent cases with rifampicin resistance (1 case) and isoniazid resistance (2 cases) were attributed to endogenous relapse. The comparison of the characteristics between endogenous reactivation and exogenous reinfection by Fisher's exact test were shown in Table 4. The *P-*value of these factors was >0.05 with no significant difference.

**Table 4:** Comparison of the characteristics between endogenous reactivation and exogenous reinfection.

**Factors**		***N***	**Endogenous reactivation**		**Exogenous reinfection**		***P*** -value^*^
			***n***	**%**	***n***	**%**	
Sex	Male	31	19	86.36	12	80.00	0.670
	Female	6	3	13.64	3	20.00	
Age (years)	<=25	4	2	9.09	2	13.33	0.611
	26–54	10	5	22.73	5	33.33	
	≥55	23	15	68.18	8	53.33	
Contact history	Yes	2	1	4.55	1	0.00	1.000
	No	35	21	95.45	14	100.00	
Smoking	Yes	15	11	50.00	4	26.67	0.190
	No	22	11	50.00	11	73.33	
Drinking	Yes	7	5	22.73	2	13.33	0.677
	No	30	17	77.27	13	86.67	
Pulmonary cavity	Yes	20	10	45.45	10	66.67	0.315
	No	17	12	54.55	5	33.33	
Beijing family	Yes	33	19	86.36	14	93.33	0.633
	No	4	3	13.64	1	6.67	
Rifampicin resistance	Yes	1	1	4.55	0	0.00	1.000
	No	36	21	95.45	15	100.00	
Isoniazid resistance	Yes	2	2	9.09	0	0.00	0.505
	No	35	20	90.91	15	100.00	

* *Fisher's exact test*.

## Discussion

In this molecular epidemiological study, we genotyped 2,098 strains circulating in Eastern China. We observed that the types of prevalent *M.tb* strains were relatively single, with the dominant strain of the Beijing family. Endogenous reactivation appeared to be a major cause of recurrent TB, emphasizing the importance of case management and long-term follow-up. Although MIRU-VNTR typing has been widely used to examine the genetic diversity and transmission of *M.tb* strains, this study has a relatively large sample size and provided robust statistical power.

We categorized *M.tb* strains into five groups, and the dominant isolates were category IV, accounting for 77.46% of all isolates. We observed that the RD105 deletion (Beijing family) contributed to the predominant infection, which was consistent with findings by Larry D. Teeter (23) and Carlo Garzelli (24). In addition, we found a significant association between non-dominant strains and pulmonary cavitation, which was consistent with the report by Lazzarini (25). In China, although the main lineage of *M*.tb is the Beijing family, non-dominant strains mainly characterized by the LAM family still keeps an important composition (26). Liu et al. reported that the Beijing family was the dominant genotype (83.3%) in Beijing and linked to the recent spread of TB (27). The Beijing family strain was also found to be the most common genotype in Shanxi (81.5%) and Yunnan (70.5%), which was related to an increased risk of MDR-TB (28, 29). In Xinjiang, the Beijing lineage strains accounted for 57.48% of total *M.tb* isolates (30).

We observed that the recurrence rate of TB was 7.43%, which was higher than the proportion reported by Xie (6.5%) (31) and slightly lower than that reported by Ahmad (32) (8.0%). Other studies in China showed that the recurrence rate was 7.6% (33) or 6.9% (34). A prospective study and paired analysis emphasized that endogenous reactivation (62.16%) was the leading cause of recurrent TB in Eastern China, which was lower than that in India (35) (93%) and Australia (36) (87%). Endogenous reactivation mainly occurred in high-burden countries and was related to Beijing family genotype (9), infection with drug-resistant TB (37), low socioeconomic status, and foreign birth (38). Previous studies have shown that most recurrent TB cases in HIV uninfected patients were due to endogenous reactivation, while most TB recurrence in HIV-infected patients was exogenous reinfection (39, 40). In our study, we found that the proportion of males and patients with tobacco smoking history were at a higher risk of endogenous reactivation. Patients with MDR, rifampicin resistance, and isoniazid resistance were related to an increased risk of endogenous reactivation, indicating a potential correlation with antituberculosis drug resistance (37, 41).

At present, a variety of genotyping methods have been developed, such as IS6110-RFLP, Spoligotyping, MIRU-VNTR, and WGS. Although the gold standard for TB genotyping is IS6110-RFLP, it is labor-intensive, and the interpretation of data from this method can be susceptible to errors (42). Although the 15- and 24-loci MIRU-VNTR has a greater discriminatory power (42, 43), the 12-loci MIRU-VNTR can trace the recent outbreak and identify dominant isolates, requiring less time and labor (19, 44). To distinguish the endogenous reactivation and exogenous reinfection, 12-loci MIRU-VNTR is an alternative methodological choice with easy-to-apply, cost-effective and good performance. Comparing the consistency of strains isolated from paired recurrent cases can clarify the type of recurrence and take targeted clinical intervention measures (44, 45).

In this study, we used the RD105 deletion to identify the Beijing family strains. There were 1,727 (82.32%) strains being judged as Lineage 2 Beijing genotype. Previous studies have assumed that Beijing family strains were more likely to develop into MDR-TB (46). Moreover, men and older adults were also observed to be more susceptible to Beijing genotype infection (27). Using MIRU-VNTR and detection of the deletion of RD105 synthetically provides a simple, fast, and effective method for identification of *M.tb* genotyping in China (47). The Beijing family genotype obtained by the RD105 test is also mutually confirmed with the reactivation cases by 12-loci MIRU-VNTR.

Several limitations should not be ignored to explain our findings. Firstly, MIRU-VNTR genotyping is usually done by amplifying a panel of 12, 15, or 24 loci. Although the 12 loci method has a low workload and was proved to have good discrimination in the Chinese population, some scientists still recommend using the standard MIRU-15 and MIRU-24, which have considerably increased discriminatory power. Secondly, we used the MIRU-VNTR to determine the type of recurrence. However, the microevolution and mutation of the strain may lead to misjudgment of exogenous reinfection and endogenous reactivation. In the next step, it is necessary to sequence the strains to identify the common mutation sites and accurately distinguish the recurrence type.

## Conclusion

The genotypes of *M.tb* strains prevalent in Eastern China are relatively single. Beijing family strain was the dominant strain. Endogenous reactivation was the leading cause of recurrent TB. Endogenous reactivation appeared to be a major cause of recurrent TB in Eastern China. This finding emphasizes the importance of case follow-up and monitoring after the completion of antituberculosis treatment.

## Data Availability Statement

The original contributions presented in the study are included in the article/Supplementary Material, further inquiries can be directed to the corresponding author/s.

## Ethics Statement

The studies involving human participants were reviewed and approved by Ethics Committee of Nanjing Medical University. Written informed consent to participate in this study was provided by the participants’ legal guardian/next of kin.

## Author Contributions

BQ and BT: conceptualization, methodology, and software. JW: validation, writing—review and editing, and funding acquisition. BQ, BT, and QL: formal analysis and visualization. QL, ZL, HS, DT, JW, ZW, and MZ: investigation and data curation. QL and WL: resources. BQ, BT, QL, ZL, HS, DT, JW, ZW, MZ, and WL: writing—original draft preparation. WL and JW: supervision and project administration. All authors have given final approval of the version to be published and agreed on the journal to which the article has been submitted.

## Funding

This study was funded by the National Natural Science Foundation of China (81973103), National Key R&D Program of China (2017YFC0907000), Medical Research Project of Jiangsu Health Commission (ZDB2020013), and Priority Academic Program Development of Jiangsu Higher Education Institutions (PAPD). The funding agencies had no role in the study design, data collection, analysis, decision to publish, or preparation of the manuscript.

## Conflict of Interest

The authors declare that the research was conducted in the absence of any commercial or financial relationships that could be construed as a potential conflict of interest.

## Publisher's Note

All claims expressed in this article are solely those of the authors and do not necessarily represent those of their affiliated organizations, or those of the publisher, the editors and the reviewers. Any product that may be evaluated in this article, or claim that may be made by its manufacturer, is not guaranteed or endorsed by the publisher.
