# Supplementary material for: A Prospective Cohort Study on the Prevalent and Recurrent Tuberculosis Isolates Using the MIRU-VNTR Typing
Source: Front Med (Lausanne). 2021 Sep 14;8:685368. doi: 10.3389/fmed.2021.685368 (PMC8476766; doi:10.3389/fmed.2021.685368)
Supplement: Supplementary file 1 [file Table_1.DOCX]

Table S1. Primers specific for sequences flanking the MIRU units

| Locus | primer sequence(5^’^-3^’^) |
| --- | --- |
| MIRU-2 F | TGGACTTGCAGCAATGGACCAACT |
| MIRU-2 R | TACTCGGACGCCGGCTCAAAAT |
| MIRU-4 F | GTCAAACAGGTCACAACGAGAGGAA |
| MIRU-4 R | CCTCCACAATCAACACACTGGTCAT |
| MIRU-10 F | GTTCTTGACCAACTGCAGTCGTCC |
| MIRU-10 R | GCCACCTTGGTGATCAGCTACCT |
| MIRU-16 F | TCGGTGATCGGGTCCAGTCCAAGTA |
| MIRU-16 R | CCCGTCGTGCAGCCCTGGTAC |
| MIRU-20 F | TCGGAGAGATGCCCTTCGAGTTAG |
| MIRU-20 R | GGAGACCGCGACCAGGTACTTGTA |
| MIRU-23 F | CTGTCGATGGCCGCAACAAAACG |
| MIRU-23 R | AGCTCAACGGGTTCGCCCTTTTGTC |
| MIRU-24 F | CGACCAAGATGTGCAGGAATACAT |
| MIRU-24 R | GGGCGAGTTGAGCTCACAGAA |
| MIRU-26 F | TAGGTCTACCGTCGAAATCTGTGAC |
| MIRU-26 R | CATAGGCGACCAGGCGAATAG |
| MIRU-27 F | TCGAAAGCCTCTGCGTGCCAGTAA |
| MIRU-27 R | GCGATGTGAGCGTGCCACTCAA |
| MIRU-31 F | ACTGATTGGCTTCATACGGCTTTA |
| MIRU-31 R | GTGCCGACGTGGTCTTCAT |
| MIRU-39 F | CGCATCGACAAACTGGAGCCAAAC |
| MIRU-39 R | CGGAAACGTCTACGCCCCACACAT |
| MIRU-40 F | GGGTTGCTGGATGACAACGTGT |
| MIRU-40 R | GGGTGATCTCGGCGAAATCAGATA |

Table S2. PCR reaction system

| Component | PCR reaction system(μl) |
| --- | --- |
| 2*Taq PCR MasterMIX | 6 |
| Upper primers | 0.5 |
| Lower primers | 0.5 |
| DNA template | 2 |
| DDH_2_O | 3 |
| total capacity | 12 |

Table S3. The copy numbers of 37 paired recurrent strains and Beijing family genotype obtained by RD105 test

| ID | MIRU2 | MIRU4 | MIRU10 | MIRU16 | MIRU20 | MIRU24 | MIRU26 | MIRU27 | MIRU31 | MIRU39 | MIRU40 | Beijing Family detected by RD105 test * |
| --- | --- | --- | --- | --- | --- | --- | --- | --- | --- | --- | --- | --- |
| 01-1 | 2 | 1 | 3 | 3 | 2 | 1 | 7 | 3 | 6 | 3 | 3 | 1 |
| 01-2 | 2 | 2 | 3 | 3 | 2 | 1 | 7 | 3 | 5 | 2 | 3 | 1 |
| 02-1 | 2 | 2 | 3 | 3 | 2 | 1 | 7 | 3 | 5 | 3 | 3 | 1 |
| 02-2 | 2 | 2 | 3 | 3 | 2 | 1 | 7 | 3 | 5 | 3 | 3 | 1 |
| 03-1 | 2 | 2 | 3 | 3 | 2 | 1 | 7 | 3 | 5 | 3 | 3 | 1 |
| 03-2 | 2 | 2 | 3 | 3 | 2 | 1 | 7 | 3 | 5 | 3 | 3 | 1 |
| 04-1 | 2 | 2 | 2 | 3 | 2 | 1 | 7 | 3 | 5 | 3 | 3 | 1 |
| 04-2 | 2 | 2 | 2 | 3 | 2 | 1 | 7 | 3 | 5 | 3 | 3 | 1 |
| 06-1 | 2 | 2 | 3 | 3 | 2 | 1 | 7 | 3 | 5 | 3 | 3 | 1 |
| 06-2 | 2 | 2 | 3 | 3 | 2 | 1 | 7 | 3 | 5 | 3 | 3 | 1 |
| 07-1 | 2 | 2 | 3 | 3 | 2 | 1 | 6 | 3 | 5 | 3 | 3 | 1 |
| 07-2 | 2 | 2 | 3 | 3 | 2 | 1 | 6 | 3 | 5 | 3 | 3 | 1 |
| 08-1 | 2 | 2 | 3 | 3 | 2 | 1 | 7 | 3 | 4 | 3 | 3 | 1 |
| 08-2 | 2 | 2 | 3 | 3 | 2 | 1 | 7 | 3 | 4 | 3 | 3 | 1 |
| 09-1 | 2 | 2 | 3 | 3 | 2 | 1 | 7 | 3 | 5 | 3 | 3 | 1 |
| 09-2 | 2 | 2 | 3 | 3 | 2 | 1 | 7 | 3 | 5 | 3 | 3 | 1 |
| 10-1 | 2 | 2 | 3 | 3 | 2 | 1 | 6 | 3 | 6 | 3 | 3 | 1 |
| 10-2 | 2 | 2 | 3 | 3 | 2 | 1 | 7 | 3 | 6 | 3 | 3 | 1 |
| 11-1 | 2 | 5 | 2 | 3 | 2 | 1 | 5 | 2 | 2 | 2 | 2 | 0 |
| 11-2 | 2 | 5 | 2 | 3 | 2 | 1 | 5 | 2 | 1 | 2 | 2 | 0 |
| 12-1 | 2 | 2 | 2 | 3 | 2 | 1 | 4 | 3 | 3 | 2 | 3 | 0 |
| 12-2 | 2 | 2 | 2 | 3 | 2 | 1 | 4 | 3 | 1 | 2 | 3 | 0 |
| 14-1 | 2 | 2 | 3 | 3 | 2 | 1 | 6 | 3 | 5 | 3 | 3 | 1 |
| 14-2 | 2 | 2 | 3 | 3 | 2 | 1 | 6 | 3 | 5 | 3 | 3 | 1 |
| 15-1 | 2 | 2 | 3 | 3 | 2 | 1 | 6 | 3 | 5 | 3 | 3 | 1 |
| 15-2 | 2 | 2 | 3 | 3 | 2 | 1 | 6 | 3 | 5 | 3 | 3 | 1 |
| 16-1 | 2 | 2 | 3 | 2 | 2 | 1 | 7 | 3 | 5 | 3 | 3 | 1 |
| 16-2 | 2 | 2 | 3 | 2 | 2 | 1 | 7 | 3 | 4 | 3 | 3 | 1 |
| 17-1 | 2 | 4 | 2 | 3 | 2 | 1 | 5 | 3 | 3 | 2 | 2 | 0 |
| 17-2 | 2 | 3 | 2 | 3 | 2 | 1 | 5 | 2 | 3 | 2 | 2 | 0 |
| 18-1 | 2 | 2 | 3 | 3 | 2 | 1 | 7 | 3 | 5 | 3 | 3 | 1 |
| 18-2 | 2 | 2 | 3 | 3 | 2 | 1 | 7 | 3 | 5 | 3 | 3 | 1 |
| 19-1 | 2 | 2 | 3 | 3 | 2 | 1 | 7 | 3 | 5 | 2 | 3 | 1 |
| 19-2 | 2 | 2 | 3 | 3 | 2 | 1 | 7 | 3 | 5 | 2 | 3 | 1 |
| 20-1 | 2 | 2 | 3 | 3 | 2 | 1 | 6 | 3 | 5 | 3 | 3 | 1 |
| 20-2 | 2 | 2 | 3 | 3 | 2 | 1 | 6 | 3 | 5 | 3 | 3 | 1 |
| 21-1 | 2 | 2 | 1 | 2 | 2 | 1 | 7 | 3 | 5 | 3 | 3 | 1 |
| 21-2 | 2 | 2 | 1 | 1 | 2 | 1 | 7 | 3 | 5 | 3 | 3 | 1 |
| 23-1 | 2 | 2 | 4 | 3 | 2 | 1 | 5 | 4 | 6 | 3 | 3 | 1 |
| 23-2 | 2 | 2 | 3 | 3 | 2 | 1 | 5 | 3 | 5 | 3 | 3 | 1 |
| 26-1 | 2 | 2 | 2 | 3 | 2 | 1 | 7 | 3 | 5 | 3 | 3 | 1 |
| 26-2 | 2 | 2 | 2 | 3 | 2 | 1 | 7 | 3 | 5 | 3 | 3 | 1 |
| 27-1 | 2 | 2 | 3 | 3 | 2 | 1 | 7 | 3 | 5 | 3 | 3 | 1 |
| 27-2 | 2 | 2 | 3 | 3 | 2 | 1 | 7 | 3 | 5 | 3 | 3 | 1 |
| 28-1 | 2 | 2 | 3 | 3 | 2 | 1 | 7 | 3 | 5 | 3 | 3 | 1 |
| 28-2 | 2 | 2 | 3 | 3 | 2 | 1 | 7 | 3 | 5 | 3 | 3 | 1 |
| 29-1 | 2 | 2 | 3 | 3 | 2 | 1 | 7 | 3 | 5 | 3 | 3 | 1 |
| 29-2 | 2 | 2 | 3 | 1 | 2 | 1 | 6 | 3 | 3 | 2 | 4 | 0 |
| 30-1 | 2 | 2 | 3 | 3 | 2 | 1 | 3 | 3 | 5 | 3 | 3 | 1 |
| 30-2 | 2 | 2 | 3 | 3 | 2 | 1 | 3 | 3 | 5 | 3 | 3 | 1 |
| 31-1 | 2 | 2 | 3 | 3 | 2 | 1 | 7 | 3 | 3 | 2 | 4 | 1 |
| 31-2 | 2 | 2 | 3 | 3 | 2 | 1 | 5 | 3 | 5 | 3 | 3 | 1 |
| 32-1 | 2 | 2 | 3 | 3 | 2 | 1 | 7 | 3 | 5 | 3 | 3 | 1 |
| 32-2 | 2 | 2 | 3 | 3 | 2 | 1 | 5 | 3 | 5 | 3 | 3 | 1 |
| 33-1 | 2 | 2 | 3 | 3 | 2 | 1 | 7 | 3 | 5 | 3 | 3 | 1 |
| 33-2 | 2 | 2 | 3 | 3 | 2 | 1 | 7 | 3 | 5 | 3 | 3 | 1 |
| 34-1 | 2 | 2 | 3 | 3 | 2 | 1 | 7 | 3 | 5 | 3 | 3 | 1 |
| 34-2 | 2 | 2 | 3 | 2 | 2 | 1 | 7 | 3 | 5 | 3 | 3 | 1 |
| 35-1 | 2 | 2 | 3 | 3 | 2 | 1 | 7 | 3 | 5 | 3 | 3 | 1 |
| 35-2 | 2 | 2 | 2 | 3 | 2 | 1 | 0 | 0 | 5 | 3 | 3 | 1 |
| 36-1 | 2 | 2 | 3 | 3 | 2 | 1 | 8 | 3 | 5 | 4 | 3 | 1 |
| 36-2 | 2 | 2 | 2 | 3 | 2 | 1 | 7 | 3 | 5 | 3 | 3 | 1 |
| 37-1 | 2 | 2 | 3 | 3 | 2 | 1 | 7 | 3 | 5 | 3 | 3 | 1 |
| 37-2 | 2 | 4 | 2 | 3 | 2 | 1 | 3 | 2 | 3 | 2 | 3 | 0 |
| 39-1 | 2 | 4 | 2 | 3 | 2 | 1 | 4 | 2 | 3 | 2 | 3 | 0 |
| 39-2 | 2 | 4 | 2 | 3 | 2 | 1 | 5 | 2 | 3 | 2 | 3 | 0 |
| 40-1 | 2 | 2 | 3 | 3 | 2 | 1 | 6 | 3 | 5 | 3 | 3 | 0 |
| 40-2 | 2 | 2 | 3 | 3 | 0 | 1 | 7 | 3 | 5 | 3 | 4 | 1 |
| 41-1 | 2 | 2 | 3 | 3 | 2 | 1 | 7 | 3 | 5 | 3 | 4 | 1 |
| 41-2 | 2 | 2 | 3 | 3 | 2 | 1 | 7 | 3 | 5 | 3 | 4 | 1 |
| 43-1 | 2 | 2 | 3 | 3 | 2 | 1 | 7 | 3 | 5 | 3 | 3 | 1 |
| 43-2 | 2 | 2 | 3 | 3 | 2 | 1 | 7 | 3 | 5 | 3 | 3 | 1 |
| 46-1 | 2 | 2 | 3 | 3 | 2 | 1 | 7 | 3 | 6 | 3 | 3 | 1 |
| 46-2 | 2 | 2 | 3 | 3 | 2 | 1 | 7 | 3 | 5 | 3 | 3 | 1 |

*1 represents Beijing family strains, 0 represents non-Beijing family strains.
